# Effects of Ambient Environmental Factors on the Stereotypic Behaviors of Giant Pandas *(Ailuropoda melanoleuca)*

**DOI:** 10.1371/journal.pone.0170167

**Published:** 2017-01-20

**Authors:** He Liu, Hejun Duan, Cheng Wang

**Affiliations:** 1 Beijing Key Laboratory of Captive Wildlife Technology, Giant Panda Research Centre, Beijing Zoo, Beijing, China; 2 Beijing Municipal Key Laboratory of Food Poisoning Diagnosis Traceability Technology, Beijing Centre of Disease Control and Prevention, Beijing, China; 3 Key Laboratory of Digital Earth Science, Institute of Remote Sensing and Digital Earth, Chinese Academy of Sciences, Beijing, China; Institute of Zoology, CHINA

## Abstract

Stereotypies are commonly observed in zoo animals, and it is necessary to better understand whether ambient environmental factors contribute to stereotypy and how to affect animal welfare in zoo settings. This study investigated the relationships between stereotypic behaviors and environmental factors including ambient temperatures, humidity, light intensity, sound intensity and number of visitors. Seven giant pandas were observed in three indoor enclosures and three outdoor enclosures. Environmental factors were measured for both indoor and outdoor enclosures and the effect they had on stereotypical behaviors was investigated. Our research found that light intensity significantly correlated with all stereotypies behaviors. Higher environmental temperature reduced the duration of pacing but increased the frequency of pacing, the duration and frequency of door-directed, meanwhile the duration of head-toss. However, we found no noticeable effect of humidity on stereotypic behaviors except for the frequency of head-toss. We also found that sound intensity was not correlated with stereotypies. Finally, the growth of visitors was negatively associated with the duration of door-directed. These results demonstrated that various environmental factors can have significant effects on stereotypic behaviors causing the expression of various stereotypies. Thus, stereotypies in zoo animals may not simply represent suboptimal welfare, but rather might be adopted as a means of coping with an aversive environment.

## Introduction

Zoo environments, though they often strive to mimic natural environments, are necessarily different from an animal’s *in-situ* habitat. Stereotypic behavior is rarely observed in wild animals, which means that zoo-housed animals may reflect an abnormal interaction between animal and environment [1]. Stereotypies are often associated with a variety of possible stressors, including inadequate control over environment and lack of opportunity to express natural behavior [2, 3]. Thus, stereotypies have often been used as behavioral measures of animal welfare or psychological well-being [4, 5, 6, 7]. Although the causes of stereotypic behavior are often unknown, stereotypies have been shown to be environmentally induced, developing in situations when an animal’s life is in some way less than optimal [1, 8, 9]. In such instances, it is thought that stereotypy is a homeostatic adaptation by zoo-housed animals to cope with their environment [10, 11, 12]. It is also suggested that stereotypic behavior may be one method by which captive animals pass their time, or use it as a substitute for normal free-ranging behavior [13, 14]. Furthermore, some authors have demonstrated that stereotypies have evolved as a kind of self-generated enrichment [4, 15].

Stereotypic behavior has been well documented in zoo-housed animals [16, 17, 18, 19, 20, 21, 22, 23, 24, 25]. Several environmental factors, including space limitation [26, 27], effects of feeding schedule [1, 28], restraint of environmental control [29], and lack of stimulation [6, 30], have been identified in relation to the development of stereotypies [31, 32]. Zoo-housed carnivores generally exhibit stereotypic pacing [33]. For example, leopard cats (*Felis bengalensis*) housed near natural predators or provided with inadequate hiding spaces, increased their stereotypic pacing [2]. Similarly, stereotypical pacing in leopards (*Panthera pardus*) also increased when kept “off exhibit” in small, barren holding quarters devoid of natural light [34].

Other studies have found that there were great interspecies differences in terms of the types of stereotypies which zoo-housed animal might develop. Farmed blue foxes (*Alopex lagopus*) increased locomotory stereotypic behaviors, when they were provided with larger cages [26]. Alternatively, walruses (*Odobenus rosmarus*) and giraffes (*Giraffa camelopardalis*) often display an oral stereotypic behavior [16, 35–37]. Even closely related animals often differ in their behavioral responses to zoo environments [1]. Polar bears (*Ursus maritimus*) express stereotypic head swing, stereotypic walking and repetitive swimming bouts in a zoo setting [27, 35]. Asiatic black bears (*Ursus thibetanus*) and Malayan sun bears (*Helarctos malayanus*) have been observed to exhibit locomotor, oral and head swaying stereotypies [36–38]. Mammals are not the only species to express stereotypies and different species develop different stereotypic behaviors [39]. Cockatoos are very prone to self-plucking, whereas certain other parrot species are far less prone [40].While previous research has explored the relationship between a single ambient environmental factor and stereotypical behaviors [41], there is a general lack of information on how zoo-housed animals cope with variations to a suite of different environmental factors. Rees [41] described how zoo-housed Asian elephants (*Elephas maximus*) exhibited a strong negative correlation between maximum daily temperature and the frequency of stereotypic behavior. Carlstead [10] reported that the time spent pacing in fennec foxes (*Vulpes zerda*) was linked to the number of zoo visitors. Owen et al. [42] and Powell et al. [43] analyzed the effect of construction noise on giant pandas and found that noise was associated with changes in behavior. Very few studies report the influence of multiple environmental factors, such as ambient temperature, humidity, sound intensity, light intensity and visitors on the stereotypic behaviors within a zoo environment. It is also unclear if specific environmental factors induce certain stereotypies or if they cause a general increase.

As an endangered species, giant pandas represent a symbol of animal conservation, becoming quite common in worldwide zoos, with 396 individuals distributed within 72 zoos in 15 countries including the United states, Canada, Mexico, United Kingdom, Austria, Belgium, France, Spain, Australia, Japan, Malaysia, Singapore and Thailand in 2014 [44, 45, 46]. For the well-being of species kept in zoos outside of their natural habitat, including but not limited to the giant panda, it is crucial to learn the relationship between ambient environmental factors of zoos and stereotypic behaviors. However, there is a lack of specific studies on ambient environmental conditions and their relationship to stereotypies.

The objectives of the current study on giant panda are as follows: a) to compare the duration and frequency of stereotypic behaviors exhibited by giant pandas in both outdoor and indoor enclosures; b) to determine whether stereotypic behaviors are influenced by ambient environmental factors; c) to analyze the relationship between stereotypy and animal welfare.

## Material and Methods

### Ethics Statement

No special permission for use of animals in observational behavioral studies is required in China. This study was approved by the faculty of the Beijing zoo and National Bureau of Forestry. The individual in this manuscript has given written informed consent (as outlined in PLOS consent form) to publish case details of this study.

### Study Animals and Husbandry

The seven zoo-housed giant pandas in the study were born in captivity and held individually by the Beijing zoo (Table 1). The giant pandas alternate between inside enclosures and outside exhibition areas every two days. Pandas were moved outside between 08:00 and 08:30 and were returned to indoor enclosures between 16:00 and 17:00. Behavioral observation sessions did not include the time period of 11:00–14:00 because zoo-housed giant pandas usually rest during this time frame and are largely inactive[47, 48, 49]. Details of the three large outside exhibitions and three small indoor enclosures are presented in Table 2.

**Table 1:** Biographical Information of the Giant Pandas.

Name	Studbook number	Gender	Birth (MM/DD/YY)	Origin
Gugu	496	Male	09/25/1999	Born in Wolong
Dadi	394	Male	09/22/1992	Born in Wolong
Jini	403	Female	11/04/1993	Born in Beijing
Yinghua	566	Female	08/17/2003	Born in Wolong
Meng	652	Female	09/13/2006	Born in Wolong
Lele	320	Female	09/08/1986	Born in Beijing
Niu Niu	421	Female	09/05/1999	Born in Beijing

**Table 2:** Key Differences in Enclosure Characteristics for Outdoor and Indoor.

Outdoor Enclosure	Indoor Enclosure
Large Area (950m^2^, 550 m^2^, 450 m^2^)	Small Area (120m^2^, 70m^2^, 50m^2^)
Natural substrates, grass, bushes, trees, logs	Concrete floor, stones, glass and walls
Ambient temperature	Generally cooler in summer and warmer in winter
Nature light	Artificial light
Exposed to ambient noise	Noise buffered by glass

Note: we obtained the size of enclosures from an architectural plan of the giant panda house.

Bamboo and water were supplied to the giant pandas at various times of day at every enclosure as their main diet. Supplementary diets containing small amounts of apple, carrot, egg, beef and specialized biscuit, were fed to the pandas twice daily between 08:30–09:30 and 15:30–16:30 at every enclosure. In order to minimize interference from keepers during the observation period, the feeding time was delayed in the afternoon until the end of the observation.

### Behavioral Data Collection

An ethogram with four categories of stereotypic behavior is listed in Table 3[48]. At the beginning of the formal observation, there was a pilot observation allowing observers to become familiar with giant pandas behavior. After a brief training session, interobserver agreement was determined based on observers scoring the same 10 mins video of giant panda behavior. Interobserver reliability was greater than or equal to 95% [50, 51, 52]. Focal sampling and continuous recording methods were taken during observation [53]. Observations were conducted over two periods (9:00–11:00 and 14:00–16:00) daily from 1^st^ February to10^th^ May. One giant panda was observed for each period.

**Table 3:** Ethogram of Giant Panda stereotypical Behavior.

Stereotypic behaviors	Symbol	Description
Pacing	PC	Back and forth, or perimeter traveling in a repetitive, sustained, stereotyped pattern. Must travel the same route at least 3 times.
Head-toss	HT	Animal abruptly lifting head upward and/or to the side in a swinging movement, often occurs during turning. These actions are repeated at least 3 times.
Head-weaving	HE	Standing in one place and continuously moving the head from side to side horizontally at least 3 times.
Door-directed	DR	Animal waits at the door with restlessness. Standing on hind legs and putting fore legs on door to knock or push the door, or scratching itself to indicate an anticipatory of food or keepers. These actions are repeated at least 3 times in succession.

Giant pandas were randomly observed on an individual basis in different enclosures. Observation lasted 4 hours with five different environmental factors being measured each day. The observers were located on the outside perimeter of enclosures to ensure visual access to pandas during the entirety of the recording period [53].

All behavioral data were recorded as duration and frequency of occurrence. The duration of behavior was the percentage of time spent on performing the behavior within one hour[48, 49]. The frequency of behavior was the number of times that behavior occurred per hour[48, 49]. The different observations in the indoor versus outdoor were considered as statistically independent and multiple observations from the same individual in one hour were considered as statistically independent of one another hour [54].

### Environmental Data Collection

Ambient temperature and humidity were measured using a TES 1360A digital humidity/ temperature meter (TES Electrical Electronic Corp, Taiwan). Light intensity was collected by a TES 1335 digital light meter (TES Electrical Electronic Corp. Taiwan). Temperature, humidity and light intensity were examined at 09:00, 10:00, 11:00, 14:00, 15:00 and 16:00. The temperature, humidity and light intensity were calculated by averaging each measurement per hour. Ambient sound was measured using a TES-1351 sound level meter (TES Electrical Electronic Corp. Taiwan) inside and outside of the enclosures. A low range measurement of 35–90 dB(decibel units) and A-weighting were used to obtain a slow response and comparatively stable sound level of giant panda house. The sound intensity was recorded in decibels at ten-minute intervals and averaged all measurement per hour. The number of visitors were recorded by counting the total number of people around the subject’s enclosures in each hour.

### Statistical Analysis

The conventional statistics are applied for the effects of environmental measurements on each individual test. Total seven giant pandas were observed and then each giant panda was examined independently, the descriptive statistics are reported.

PASW Statistics software for Windows (version 17.0, SPSS Inc.: Chicago, IL, USA) was applied in the statistical analysis. All data were determined for assumption of normality by one-sample Kolmogorov-Smirnov test and homogeneity of variance by Levene’s test. The duration and frequency of each behavior were calculated for one hour observation period. All data including environmental factors and behaviors were tested to be non-normally distributed, thus non-parametric data was transformed by arcsine, square root or logarithm. However, for most data were still non-parametric after transformation, non-parametric analysis was then used to analyze all data. Generalized Linear Models (GLMs) were conducted to examine the relationship between behavior and environmental factors. Mann-Whitney U test was used to compare the environmental factors between indoor enclosure and outdoor enclosure. Nonparametric Kruskal-Wallis tests were used to determine the individual difference within samples. There were no significant differences in the stereotypic behaviors based on gender and age (P>0.05). Wilcoxon signed ranks test was performed to compare the difference of behaviors and environmental variables between indoor enclosures and outdoor enclosures. The Mean±SE was reported for the untransformed data. All values (P<0.05) are presented in the text.

## Results

### Differences between Outdoor Enclosures and Indoor Enclosures

**Fig 1:**
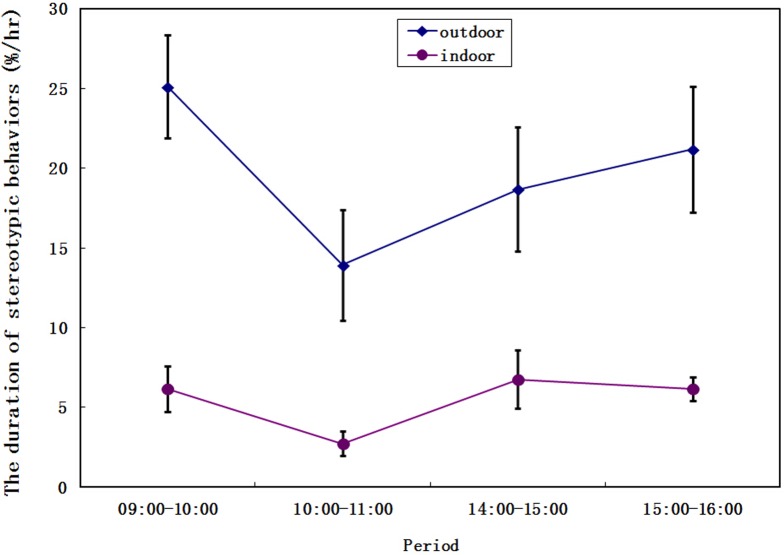
The duration of stereotypies in different periods between outdoor enclosures and indoor enclosures. Standard errors are represented in the figure by the error bars attached to each sign. Diamonds represent outdoor enclosures, circles represent indoor enclosures.

**Fig 2:**
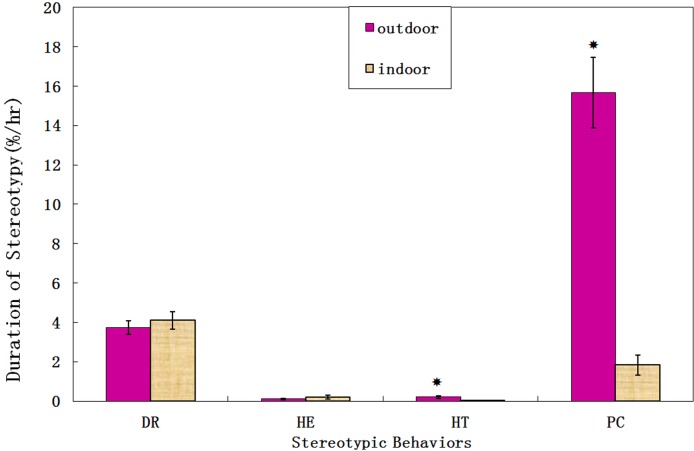
The duration of stereotypies between outdoor enclosures and indoor enclosures. The dark column represents the mean duration of stereotypies in outdoor enclosures. The wall column respresents the mean duration of stereotypies in indoor enclosures. Standard errors are represented in the figure by the error bars attached to each column. There was a significant difference in the duration for head-toss and pacing between outdoor enclosures and indoor enclosures(*P*<0.05).

**Fig 3:**
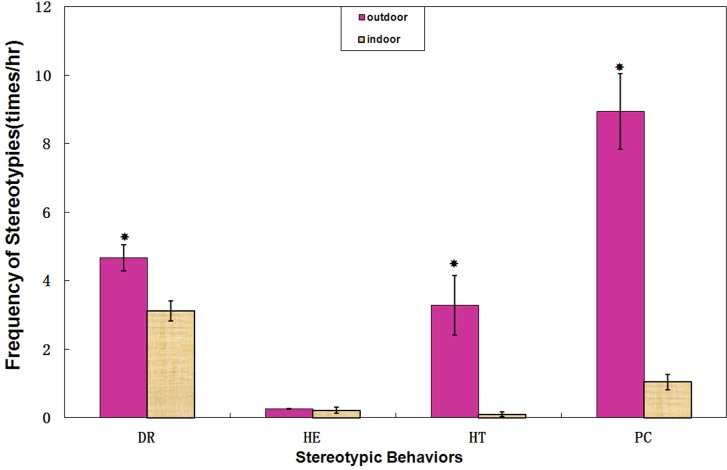
The frequency of different stereotypy in outdoor enclosures and indoor enclosures. The dark column represents the proportion of stereotypies in outdoor enclosures. The wall column respresents the proportion of stereotypies in indoor enclosures. Standard errors are represented in the figure by the error bars attached to each column. There was a significant difference in the frequency of Door-direct, Head-toss and Pacing between outdoor enclosures and indoor enclosures(*P*<0.05).

More than 522 hours of data were collected from seven giant pandas in five months, with each panda being observed for seventy hours on average. The sum of duration of all four stereotypies (Pacing, Head-toss, Head-weaving, and Door-directed) between outdoor and indoor enclosures exhibited similar trends in different periods (Fig 1). The duration and frequency of stereotypic behaviors in outdoor enclosures were significantly higher than those in indoor enclosures (Z = -5.381, *P*<0.001; Z = -6.651, *P*<0.001; respectively). Particularly, giant pandas showed significantly higher duration (Z = -5.737, *P*<0.001; Z = -7.477, *P*<0.001; respectively, Fig 2) and frequency (Z = -5.697, *P*<0.001; Z = -7.550, *P*<0.001; respectively, Fig 3) for head-toss and pacing in outdoor enclosures. By contrast, there were no significant differences in the duration for door-directed and head-weaving (Z = -0.498, *P* = 0.619; Z = -0.869, *P* = 0.385; respectively; Fig 2), but the frequency of door-directed in outdoor environment was higher than those for indoor enclosures (Z = -3.557, *P*<0.001, Fig 3).

There was a significant difference in environmental factors between indoor enclosures and outdoor enclosures (Table 4). Light intensity of outdoor enclosures was significantly higher than that of indoor enclosures (Z = -19.652, *P*<0.001, Table 4). However, the humidity, sound intensity and number of visitors for external enclosures were significantly lower than those for internal enclosures (Z = -3.392, *P* = 0.001; Z = -11.973, *P*<0.001; Z = -2.440, *P* = 0.015; respectively, Table 4). Temperature of the indoor enclosures was higher than that of the outdoor enclosures from February to mid-March, but lower from mid-March to May. However, there was no significant temperature difference between both enclosures (Z = -1.771, *P* = 0.077).

**Table 4:** Environmental Factors between Outdoor and Indoor Enclosures.

Exogenous factors		Outdoor enclosure			Indoor enclosure	
	N	Mean ± SD	Range	N	Mean ± SD	Range
Temperature (℃)	276	15.64 ±7.93	-2.38–29.20	241	16.47±2.94	7.48–22.80
Humidity (%RH)*	276	32.70 ±11.41	10.95–71.00	241	37.86 ±16.36	10.58–85.50
Light intensity (Lux)*	279	25363.00 ±13866.10	18.50–70016.67	243	68.63 ±50.37	11.20–267.45
Sound intensity (dB)*	274	56.49 ±4.22	46.87–69.20	237	62.24 ± 5.13	50.02–79.20
Number of visitors*	216	157.40±8.83	3–917	183	196.44 ±11.92	21–975

Note: “N” represents the sample size.

* indicate significant differences between indoor and outdoor enclosures *P*<0.05.

## The relationship of Environmental Factors and Stereotypies

In this study, environmental temperature ranged between -2.38°C and 29.20°C (Table 4). Ambient temperature negatively related with duration of pacing, but positively correlated with the duration of head-toss, the duration of door-directed, the frequency of door-directed and the frequency of pacing (*P*<0.05, Table 5). By contrast, ambient temperature did not significantly affect the other stereotypic behaviors such as the duration of head-weaving, the frequency of head-weaving and head-toss. Environmental humidity varied between 10.58% RH and 85.50% RH with a mean of 35.10% RH (Table 4). Pacing, door-directed, head-weaving and the duration of head-toss did not significantly correlate with RH. (*P*>0.05, Table 5).

**Table 5:** Multivariable effects analysis by GLM for Environmental Factors and Behavior in Zoo-housed Giant Pandas.

		Temperature(n = 517)		Humidity (n = 517)		Light intensity(n = 522)		Sound intensity(n = 511)		Number of visitors(n = 399)	
		Duration of time	Frequency	Duration of time	Frequency	Duration of time	Frequency	Duration of time	Frequency	Duration of time	Frequency
Pacing	Wald chi-square	**23.186**	31.342	7.588	2.084	**34.486**	37.691	0.501	0.277	1.989	2.718
	P	**0.000**	0.000	0.108	0.720	**0.000**	0.000	0.919	0.964	0.738	0.606
Head-toss	Wald chi-square	10.812	8.366	6.290	13.097	13.683	13.256	2.389	1.466	4.063	2.535
	P	0.029	0.079	0.179	0.011	0.008	0.010	0.496	0.690	0.398	0.638
Head-weaving	Wald chi-square	0.541	2.347	4.541	6.944	4.220	**11.191**	1.982	3.735	0.541	1.646
	P	0.969	0.672	0.338	0.139	0.377	**0.024**	0.576	0.292	0.969	0.800
Door-directed	Wald chi-square	10.177	12.370	5.279	7.140	**21.772**	**24.075**	2.727	1.769	**10.110**	2.284
	P	0.038	0.015	0.260	0.129	**0.000**	**0.000**	0.436	0.622	**0.039**	0.684

Note: Bold indicate that the stereotypies were significantly negatively correlated with environmental factors (*P*<0.05). The duration of pacing negatively correlated with temperature and light intensity. The duration of head-toss positively correlated with temperature and light intensity, while the frequency of head-toss positively correlated with humidity and light intensity. The frequency of pacing positively correlated with temperature and light intensity; the frequency of head-weaving negatively correlated with light intensity. Door-directed positively correlated with temperature, while negatively correlated with light intensity. The duration of door-directed negatively correlated with the number of visitors

In outdoor exhibition, light intensity maximum was at midday when the sun was directly overhead at 70,016.67 Lux. The average Lux value was 25,363.00 (SD = 13,866.08; n = 279). Light intensity diminished to 11.20 Lux (SD = 50.37, n = 243) in indoor enclosures, averaging at 68.63. Head-toss, pacing and door-directed behaviors presented a strong correlation with light intensity (*P*<0.05, Table 5).

The mean sound intensity was 59.16±5.47 dB (n = 511). The maximum noise was 79.20dB and the minimum was 46.87 dB (see Table 4). The level of sound did not significantly affect the stereotypic behaviors and there were no significant correlations between number of visitors and the stereotypic behaviors (*P*>0.05, Table 5).

## Discussion

### Effect of Environmental Factors

Of the five environmental factors recorded in this study, humidity had the lowest effect on the stereotypic behavior. Nevertheless, temperature and light intensity were significantly related to stereotypic behaviors. When comparing stereotypic behaviors of giant pandas, a significant difference emerges between outdoor and indoor enclosures. Giant pandas surprisingly spent more time on pacing and head-tossing and showed a significant increase in frequency of door-directed behavior when located in the outdoor enclosures. Similar results were observed in Indian leopards (*Panthera pardus*) which displayed higher levels of stereotypic pacing in off-show exhibit areas than on-show exhibit enclosures [34]. Similarly, Tian et al.[24] found that stereotypic behaviors of juvenile giant pandas did not differ significantly between semi-natural enclosures and traditional enclosures. Our results indicate that environmental differences between outdoor versus indoor enclosures play an important role in behavioral management and husbandry of zoo-housed giant pandas.

### Different Environmental Factors Induced Different Stereotypies

Giant pandas may take an avoidance strategy to cope with anthropogenic environmental change, due to the absence of an ability to escape the potential inadequate environmental stimuli in the zoo. Ambient temperature and light intensity had a significant influence on pacing behavior. Rees [41] reported that low environmental temperature caused an increase of stereotypic behavior in zoo-housed Asian elephants. In contrast, our current study showed that higher ambient temperature caused a reduction of the duration and a rise of frequency on pacing in giant pandas. Giant pandas have thick fur and usually prefer cooler temperatures [46], but they also regulate their body temperatures to avoid thermal stress by changing their location based on microclimate [55], so elevated environmental temperatures may have increased their desire to return to their indoor enclosure for food or shelter. With an increase of light intensity, giant pandas showed the reduced door-directed behavior and the percentage of pacing, conversely an increase of head-toss behavior and the frequency on pacing. The giant panda adapted to the sound level and the number of visitors in zoos which did not influence their stereotypy. Similarly, the giant pandas in San Diego zoo and Washington National zoo were reported to be relatively unaffected by ambient noise[42, 43].

Light intensity promoted the duration and frequency of head-toss and the frequency of pacing, but declined the duration and frequency of door-directed and the duration of pacing in this study. Free-ranging giant pandas usually live in a dense bamboo forest where there is an absence of sunlight [45]. Owen et al.[55] reported that giant pandas often avoid bright lights. Thus, light conditions could be a key factor for stereotypic behaviors of giant pandas in zoo environments. When visitors became crowded, giant pandas only showed the decreased duration of door-directed. The other stereotypic behavior including pacing, head-toss and head-weaving behavior did not significantly change with increasing visitors. Similarly Mallapur and Chellam [34] revealed that the levels of stereotypic pacing for Indian leopards were not influenced by the presence of visitors, but they exhibited higher level of stereotypic pacing in small and barren enclosure devoid of natural light. These results demonstrate that environmental factors have different effects on stereotypic behaviors.

In zoo-housed animals, the cause of stereotypic behavior is multifaceted, so the function and purpose of stereotypic behaviors are controversial. When species-typical primary behavior patterns (foraging for food, searching for a mate, escaping or distancing oneself from conspecifics) can’t be performed in a zoo environment, a stereotypic behavior may occur as a result of this frustrated motivation [18]. Our results present evidence that giant pandas exhibited different stereotypies in response to variable environmental factors; the expression of some stereotypic behavior patterns was increased, while that of others was diminished. Understanding the role of environmental factors in development of stereotypic behavior is important to improve the husbandry of zoo-housed animals. The current study indicates there is much more to learn about environmental factors and stereotypy. A more complete understanding should aid in public awareness, zoo environment design and wildlife management.

### Stereotypy Cannot Simply Evaluate Welfare

In zoo-housed animals, stereotypic behaviors are often found to be idiosyncratic [32]. Stereotypies shifted in diversity and in different levels at which they were exhibited compared with their free-ranging counterparts [30, 56]. Different stereotypic behaviors were adapted by different animals for coping with the artificial environment [6, 13, 18]. Other environmental factors such as enclosure design, group composition, and rearing history have been described to have a profound influence on stereotypic behaviors [30].

Animals can normally regulate their behavior to conform to their surroundings [57, 58]. It is extremely difficult to replicate a giant panda’s natural environment in a zoo. Compared to the wild, a zoo environment is exceedingly confined, provides few places to hide, few species of bamboo for feeding, is often much louder than the wild, and is surrounded by human beings. However, the wild is not an idyllic place free from all problems. Giant pandas have evolved over millions of years, adapting to live in certain types of natural environments. Placing giant pandas in very different surroundings can elicit stereotypic behaviors. Thus, stereotyping may be a means of coping with an aversive environment. Stereotypic behavior is often defined as an abnormal behavior that involves diminished welfare [5, 6, 18]. Mason and Latham [11] have statistically analyzed that most of environments inducing stereotypies were connected with poor wellbeing. This suggests that stereotypical behaviors are often associated with diminished welfare [59].

In the current study, giant pandas performed stereotypic behaviors in different environmental conditions, suggesting that their well being is less than optimal. The San Diego zoo's giant pandas also displayed abnormal behavior in response to varying levels of noise from visitors [42]. Powell et al. [43] found that giant pandas exhibited more stereotypic behavior when demolition was occurring. However, they did not detect that the pandas experienced a significant decline in welfare. In fact, “Individual animals that perform stereotypies in suboptimal environments may well have better welfare than those that do not perform stereotypies in the same environment” [60]. Stereotypic behaviors should be a warning signal of variances in zoo environment, not the sole animal welfare index [11]. Thus, the relationship between stereotypy and welfare should be systematically investigated. One cannot simply rely on stereotypic behavior to evaluate welfare of animals.

In conclusion, zoo-housed pandas are maintained in captive environments that have a particular range of environmental variables that may not be found in wild setting. We found that some of these environmental factors influenced stereotypic behaviors. There is no simple connection between stereotypy and welfare. Further research should focus on the relationship between the evaluation of welfare and stereotypy.

## Supporting Information

S1 Fig Indoor exhibition of giant panda at Beijing zoo. (TIF)

S2 Fig Diagram of giant panda house at Beijing zoo. (PNG)

S1 Table Duration and Frequency of Stereotypic Behaviors for Individual Giant Panda. (DOCX)
