# Supplementary figures and images for: Effects of Ambient Environmental Factors on the Stereotypic Behaviors of Giant Pandas (Ailuropoda melanoleuca)
Source: PLoS One. 2017 Jan 20;12(1):e0170167. doi: 10.1371/journal.pone.0170167 (PMC5249093; doi:10.1371/journal.pone.0170167)

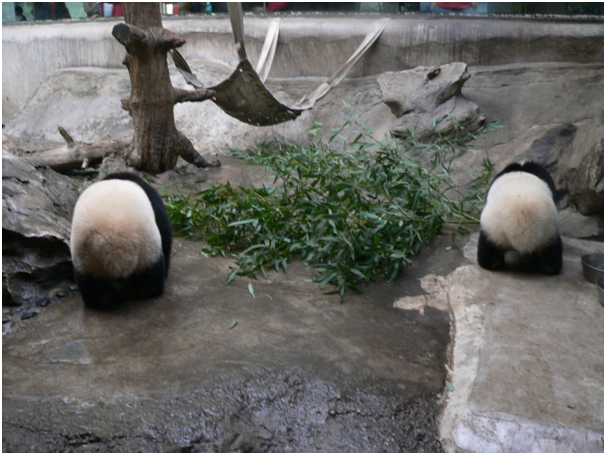

Supplement: S1 Fig — (TIF) [file pone.0170167.s001.TIF]

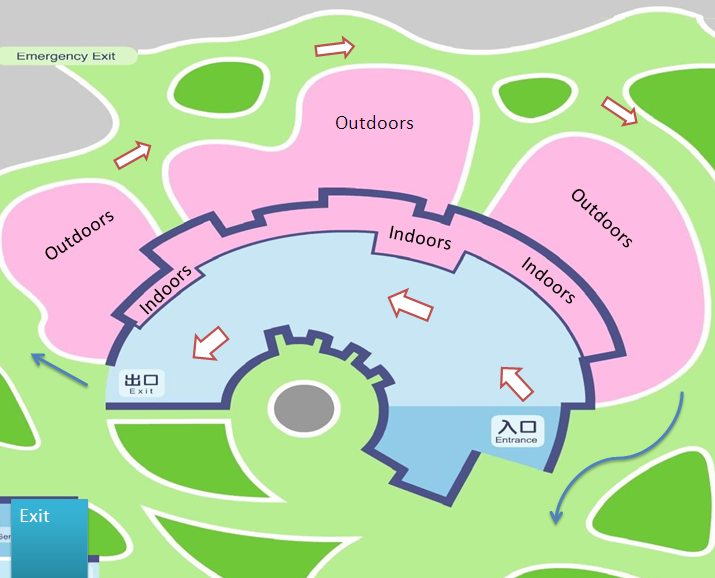

Supplement: S2 Fig — (PNG) [file pone.0170167.s002.png]
