# Supplementary material for: Effects of Ambient Environmental Factors on the Stereotypic Behaviors of Giant Pandas (Ailuropoda melanoleuca)
Source: PLoS One. 2017 Jan 20;12(1):e0170167. doi: 10.1371/journal.pone.0170167 (PMC5249093; doi:10.1371/journal.pone.0170167)
Supplement: S1 Table — (DOCX) [file pone.0170167.s003.docx]

S1 Table - Duration and Frequency of Stereotypic Behaviors for Individual Giant Panda

| Animal | N^*^ | Type | Pacing | Head-  toss | Head-  weaving | Door-  directed |
| --- | --- | --- | --- | --- | --- | --- |
| Gugu | 38  40 | Duration(%/hr) | 3.79±1.05 | 0.01±0.01 | 0.21±0.10 | 3.12±0.45 |
|  |  | Frequency(times/hr) | 2.54±0.58 | 0.03±0.02 | 0.12±0.04 | 3.78±0.54 |
| Dadi | 38  40 | Duration(%/hr) | 5.51±1.63 | 0.90±0.18 | 0.03±0.02 | 5.31±0.67 |
|  |  | Frequency(times/hr) | 6.54±1.88 | 6.16±1.71 | 0.02±0.01 | 3.45±0.40 |
| Jini | 38  37 | Duration(%/hr) | 4.88±0.97 | 3×10^-3^±2×10^-3^ | 2.39±0.76 | 4.77±0.49 |
|  |  | Frequency(times/hr) | 2.85±0.50 | 0.02±0.02 | 0.68±0.21 | 4.97±0.51 |
| Ying  hua | 38  40 | Duration(%/hr) | 19.55±2.81 | 0.58±0.27 | 0.11±0.04 | 1.59±0.26 |
|  |  | Frequency(times/hr) | 7.08±1.06 | 1.14±0.21 | 0.14±0.05 | 2.42±0.31 |
| Meng | 36  35 | Duration(%/hr) | 11.32±10.02 | 4×10^-3^±3×10^-3^ | 0.10±0.02 | 2.60±1.56 |
|  |  | Frequency(times/hr) | 3.43±2.44 | 1.62±0.38 | 0.24±0.05 | 4.00±2.42 |
| Lele | 36  35 | Duration(%/hr) | 7.94±0.87 | 0.07±0.03 | 0.12±0.07 | 2.26±0.57 |
|  |  | Frequency(times/hr) | 4.39±0.51 | 0.42±0.15 | 0.23±0.11 | 2.90±0.65 |
| Niu Niu | 36  35 | Duration(%/hr) | 3.39±2.13 | 0.42±0.11 | 0.54±0.17 | 5.25±1.73 |
|  |  | Frequency(times/hr) | 2.48±1.46 | 0.05±0.05 | 0.38±0.13 | 2.00±0.60 |

* The first number in this column is observation hours of indoor, next number is observation hours of outdoor.
